# From the national to the local: Issues of trust and a model for community-academic-engagement

**DOI:** 10.3389/fpubh.2023.1068425

**Published:** 2023-02-24

**Authors:** Olufunmilayo Chinekezi, Lauri Andress, Etsemaye P. Agonafer, Susan Massick, Sarah Piepenbrink, Karey M. Sutton, Philip M. Alberti, Desiree de la Torre, Shannon Guillot-Wright, Marshala Lee

**Affiliations:** ^1^National Academy of Sciences, Engineering, and Medicine, Washington, DC, United States; ^2^Department of Medical Education, Geisinger Commonwealth School of Medicine, Scranton, PA, United States; ^3^Department of Health System Science, Kaiser Permanente Bernard J. Tyson School of Medicine, Pasadena, CA, United States; ^4^Department of Dermatology, The Ohio State University College of Medicine, Columbus, OH, United States; ^5^AAMC Center for Health Justice, Association of American Medical Colleges, Washington, DC, United States; ^6^Health Equity Research, MedStar Health Research Institute, Washington, DC, United States; ^7^Community Affairs and Population Health Improvement, Children's National Hospital, Washington, DC, United States; ^8^Center for Violence Prevention, University of Texas Medical Branch, Galveston, TX, United States; ^9^Harrington Value Institute Community Partnership Fund, ChristianaCare, Wilmington, DE, United States

**Keywords:** health equity, community engagement, trustworthiness, healthcare, social justice

## Abstract

Inequities in health and health care in the United States have persisted for decades, and the impacts on equity from the COVID-19 pandemic were no exception. In addition to the disproportionate burden of the disease across various populations, the pandemic posed several challenges, which exacerbated these existing inequities. This has undoubtedly contributed to deeply rooted public mistrust in medical research and healthcare delivery, particularly among historically and structurally oppressed populations. In the summer of 2020, given the series of social injustices posed by the pandemic and highly publicized incidents of police brutality, notably the murder of George Floyd, the Association of American Medical Colleges (AAMC) enlisted the help of a national collaborative, the AAMC Collaborative for Health Equity: Act, Research, Generate Evidence (CHARGE)^1^ to establish a three-way partnership that would gather and prioritize community perspectives and lived experiences from multiple regions across the US on the role of academic medicals centers (AMCs) in advancing health and social justice. Given physical gathering constraints posed by the pandemic, virtual interviews were conducted with 30 racially and ethnically diverse community members across the country who expressed their views on how medical education, clinical care, and research could or did impact their health experiences. These interviews were framed within the context of the relationship between historically oppressed groups and the COVID-19 vaccine clinical trials underway. From the three-way partnership formed with the AAMC, AAMC CHARGE participants, and 30 community members from racially and ethnically diverse groups, qualitative methods provided lived experiences supporting other literature on the lack of trust between oppressed communities and AMCs. This led to the development of the Principles of Trustworthiness (PoT) Toolkit, which features ten principles inspired by community members' insights into how AMCs can demonstrate they are worthy of their community's trust^2^. In the end, the three-way partnership serves as a successful model for other national medical and health organizations to establish community engaged processes that elicit and prioritize lived experiences describing relationships between AMCs and oppressed communities.

## Introduction

The lack of public trust in medical research and healthcare delivery by historically oppressed groups is one of the most significant obstacles facing medical institutions today. Equally important are the root causes for this distrust amongst historically oppressed people, which have contributed to longstanding health and healthcare inequities (1). The COVID-19 pandemic has exacerbated these inequities and reminds us that to effectively address inequities experienced by communities that have historically been oppressed, we must first have relationships with those communities predicated on trust (2).

Academic medical centers and their partners must co-develop more authentic community-engagement strategies to build trust and ultimately facilitate effective health equity action (3). Furthermore, these strategies must acknowledge the present and historical incongruence of health and healthcare experiences between majority and minoritized groups.

In the United States, the evidence of injustices against communities of color, including those directly impacting health, supports the use of qualitative methods to study the lived experiences of these groups as they encounter healthcare systems (4, 5). The use of qualitative methods presents one of the best options for exploring whether and how structural racism, as a set of social processes and relationships, triggers biological mechanisms that impact the health of historically and structurally oppressed groups^3^. Given the value of lived experiences in elucidating social processes and issues resulting from historical and present-day structural barriers, qualitative methods are an effective pathway for developing community-academic partnerships to improve community health (6).

In 2020, the Association of American Medical Colleges (i.e., “AAMC team”) revised its 2015 process of developing annual toolkits that explore how academic medical centers engage and work with members of oppressed groups.^4^ Here, we illustrate the 2020 process used during the COVID-19 pandemic describing how the AAMC built trust across multiple regions with community members of racial and ethnic groups by working through a nationally organized third party known as AAMC Collaborative for Health Equity: Act, Research, Generate Evidence (CHARGE). By implementing community-engaged practices through an intermediary organization, AAMC was able to use qualitative methods across several local regions to successfully capture the lived experiences of community members that make up the CHARGE service areas. This three-way partnership between the national organization and multiple communities facilitated through an intermediary group ultimately produced narratives on mistrust representative of stories from historically oppressed groups and produced the toolkit entitled Principles of Trustworthiness (PoT).

The three-way partnership is a replicable and scalable model for other AMCs to elicit and prioritize widespread community insights in a virtual environment. Additionally, inspired by approaches but distinct from other existing partnership models, this process was facilitated by a national, multidisciplinary health equity network (7). This work amplifies concepts and frameworks about building the trustworthiness of AMCs among oppressed communities shared in the pre-COVID-19 literature, such as making long-term commitments, bi-directionality, and humility (8, 9). Thus, the process sets a unique precedent by establishing community-engaged practices that moved from the national level through a third-party organization down and across multiple local regions to elicit lived experiences from racial and ethnic community members that highlighted relationships between AMCs and groups that experienced historical and ongoing inequities during the COVID-19 pandemic.

## Methods

The AAMC team set the vision and goals of the initiative, identified partners that could recruit members of historically and structurally oppressed groups, and managed the logistics and coordination of all project components, including engaging all partners.

### Establishing the team and logistics

Given official in-person gathering guidelines and restrictions posed by the COVID-19 pandemic, the AAMC CHARGE, a national collaborative of health equity scholars, practitioners, and community partners, was enlisted to produce the 2021 toolkit.

The AAMC team requested applications for interested AAMC CHARGE participants (i.e., “collaborators”) with community engagement and qualitative research experience who could recruit and conduct semi-structured video interviews with local community members from across the US that had lived experiences based on membership in an oppressed group. Following the application review period, 13 collaborators were selected. Given scheduling conflicts with a few of the initially selected collaborators, the last team of collaborators consisted of nine individuals from varying organizations/institutions and regions of the country (Table 1). Before beginning the toolkit development process, the AAMC team worked to coordinate IRB clearances for some of the collaborators as required by their institutions.

**Table 1:** Geographic location of Principles of Trustworthiness Toolkit partners.

	**AAMC team**	**AAMC CHARGE collaborators**	**Community members**
Total number of participants	5	9	30
Institution/region	Washington, DC	**Baylor University**	**West:**
		Houston, Texas	Los Angeles, California
		**Children's National Hospital**	Sante Fe, New Mexico
		Washington, DC	**Midwest:**
		**Christiana Care Health System**	Columbus, Ohio
		Wilmington, Delaware	Pittsburgh, Pennsylvania
		**Fayetteville State University**	**South:**
		Fayetteville, North Carolina	New Orleans, Louisiana
		**George Washington University**	Fayetteville, North Carolina
		Washington, DC	
		**The Ohio State University**	Austin, Texas
		**Medical Center**	Galveston, Texas
		Columbus, Ohio	Houston, Texas
		**Readily Apparent**	**East:**
		Austin, Texas	Washington, DC
		**UCLA CTSI Community Engagement Research Program and Kaiser Permanente Bernard J. Tyson School of Medicine**	Wilmington, Delaware
		Los Angeles, California	
		**The University of Texas, Galveston**	
		Galveston, Texas	

A 1-h mandatory virtual training session was held for all collaborators to learn more, ask questions, and offer modifications about the project, its goals, and the process for conducting the interviews and submitting files. Each institutional team selected one person as the designated interviewer and was provided a shared Dropbox folder, which contained the following:

- Training materials for interviewers.
- Interview guide.
- Consent forms, including certified Spanish translation.
- IRB approval documentation.
- Technical configuration for optimizing and standardizing Zoom recording quality.
- Detailed Instructions for all processes.

The AAMC team chose Dropbox as a standard filesharing tool and Zoom as the preferred video recording platform due to their low cost and high accessibility across operating systems and devices.

### Community participant recruitment, interviews and analysis

While there was no universal method for recruiting interviewees, the project relied on CHARGE collaborators' extensive regional community relationships across the US. As a result, the recruitment criteria tasked CHARGE with recruiting interviewees of at least 18 years of age from racial and ethnic groups of any gender, socioeconomic status, geographic location, or educational level.

Collaboration across the national regions that came from CHARGE collaborators, geographical distances, and digital work and information environments presented unique challenges and constraints posed by COVID-19 pandemic social distancing and safety requirements. Thus, collaborators conducted most interviews virtually.

Given the potential of varying access to technology, information was provided for interviewees to sign consent forms *via* free smartphone apps such as Adobe Fill and Sign. Digital photos of signed hard-copy audio and video consent forms were also accepted and taken by either the interviewee or the collaborator. Interviewees could participate in the interview *via* smartphone, tablet, or computer *via* the free Zoom app. Collaborators used a 12-question interview guide that members of the AAMC team developed to conduct individual, semi-structured, virtual interviews with community members from each of their local regions (Supplementary material 1). The interview guide included open-ended questions to explore the community members' perspectives about how community, clinical care, medical education, and research make individuals and communities healthier. The guide was based on the 2015 interview guide co-developed by the AAMC team, the University of Maryland Medical Center, Johns Hopkins University, and their community partners. In 2015, that team sought to understand the Baltimore community's perspective on how academic medicine, across its research, clinical, and educational missions, could address local social injustice. For the 2020 iteration, we revisited that guide and shifted the focus from local injustice to the broader issue of trust in our medical and scientific institutions and communities.

After the interviews, collaborators used standardized nomenclature for saving files, uploaded all materials into their institution's respective Dropbox folder, and notified the AAMC team *via* email within 48 h of conducting an interview. A $25 gift card (either by email or a physical card sent *via* US mail, according to interviewee preference) was sent directly to the participant within 24 h of AAMC being notified. In addition, the de-identified interview audio was submitted to a transcription service. Hyperlinks to all documents (video files, separated audio tracks, consents, transcripts, and contact information for interviewees to receive gift cards) were compiled into a single spreadsheet listed by participant name. This spreadsheet was accessible only to the AAMC team to protect the interviewees' privacy.

The AAMC team used open coding to develop codes from the review of transcripts and then refined codes with a subsequent review during a series of multiple close readings during virtual meetings to discuss the key themes which emerged from the interviews (10). As part of the data analysis, the AAMC team and the collaborators selected and organized relevant interviewee quotes to appear in a professional 10-min video montage. These quotes, including their respective timestamps, were ordered and categorized according to subthemes to develop the “storyboard” for the video. The AAMC team contracted with an external pre-identified video production company to edit the interview footage accordingly. The company developed consecutive video cuts for review until a final version was approved. To ensure collaboration throughout this process, the AAMC team, collaborators, and community members remained connected through regular email communication and virtual calls, during which project updates were shared, and there were opportunities for revision. Additionally, during these exchanges, the collaborators relayed their and community members' feedback on the different cuts of the video and other components of the resulting toolkit. The AAMC team moved forward with the final products once a general consensus was met with all partners.

Simultaneously, once all community interviews and analyses were completed, the AAMC team worked with a self-selected subset of collaborators to develop a brief evaluation survey that gathered more detail about the nine collaborators' strategies to recruit interviewees and conduct their interviews. The survey was approved by AAMC and administered *via* Google Forms.

## Results

The AAMC effort resulted in the following outcomes: (1) formation of a three-way partnership between the five Association of American Medical College (AAMC) team members, nine AAMC CHARGE collaborators, and 30 diverse community members from across the nation with racial and ethnic backgrounds (Table 1) and (2) the co-development of the PoT Toolkit (Table 2, Supplementary material 2–4).

**Table 2:** Description of principles of trustworthiness toolkit components.

**Component**	**Description**
10 principles of trustworthiness	1. The community is already educated; that's why it doesn't trust you
	2. You are not the only experts
	3. Without action, your organizational pledge is only performance
	4. An office of community engagement is insufficient
	5. It doesn't start or end with a community advisory board
	6. Diversity is more than skin deep
	7. There's more than one gay bar, one “Black church,” and one bodega in your community
	8. Show your work
	9. If you're gonna do it, take your time, do it right
	10. The project may be over, but the work is not
Recorded videos and video guide	Principles of Trustworthiness Community Video, featuring footage from interviews with community members, and The Principles of Trustworthiness Orientation Video. The video guide offers suggestions for how to use each video
Interactive discussion guide	Includes pre-work for facilitators, and a series of steps for engaging audiences in interactive discussions about the Principles of Trustworthiness
Community engagement action guide	Includes a series of activities to assist in moving the 10 Principles of Trustworthiness from concept to action
Community engagement reflection guide	Includes a series of questions for personal self-reflection or as a tool to help one's organization reflect upon all 10 Principles of Trustworthiness

The AAMC CHARGE collaborators served as a liaison between the AAMC and the 30 racially and ethnically diverse local community members from regions across the US. The collaborators used snowball sampling to recruit community members *via* email and word of mouth based on previous relationships between CHARGE collaborators and specific community organizations.

The collaborators conducted 28 virtual and two in-person interviews regarding clinical trial participation in the setting of the COVID-19 vaccine trials that were underway at the time. The interviews yielded 14 h and 51 mins of video footage, with an average of 30 mins per interview. The AAMC conducted the initial data analysis and shared with all partners the primary unifying and paramount theme of trustworthiness that emerged from the interviews^5^.

For the evaluation of the partnership, nine CHARGE collaborators completed a survey that assessed their reflections and level of satisfaction with the toolkit development process and three-way partnership. Collaborators reported that it was essential to recruit participants with whom they had previously developed a trusted relationship, defined as a reliable, respectful, meaningful, and bidirectional collaboration where parties co-learn and evolve together.

Additionally, collaborators were very satisfied with the establishment, process, and final product of the three-way partnership, the PoT toolkit. Further collaborators reported satisfaction with the AAMC's vision for the initiative, facilitation of meetings, communication about recruitment logistics and conducting interviews with participants, and data analysis. Moreover, they were delighted with the way feedback and perspectives of all parties were incorporated into each stage of the initiative and reported that they were very likely to engage in future efforts of this collaboration. One collaborator stated that the “team used a completely collaborative approach that is rare to find in academic medicine […] it was a privilege to be a part of.” Collaborators thought their final product would impact academic institutions' engagement with diverse community stakeholders. Another collaborator noted, “The voice of the community partnered with the AAMC reputation will be critical in engaging medical centers to engage and learn more about this work […] I believe in the goals of the collaboration to effect change.”

## Discussion

In the end, the unique, three-way partnership is exemplary of a comprehensive approach that other AMCs can emulate to elicit and prioritize community insights and lived experiences from community members. While the initiative was conceived and led by the AAMC, we utilized a partnered approach throughout the entire process, from design to dissemination of the Principles of Trustworthiness Toolkit. Additionally, our partnership process led to the co-creation of content that adds to existing literature demonstrating why and how historically marginalized communities lack trust in academic healthcare institutions that aim to serve the public (8, 9). While the theme of distrust among marginalized communities and the 10 Principles of Trustworthiness are not novel concepts, the outcomes of this project amplify the evidence showing an increased interest in the topic during the COVID-19 pandemic. Further, the PoT Toolkit also serves to build on, support, or provide recommendations that further that trustworthiness as a foundation for effective community engagement (11, 12). Finally, guidance was provided for any organization and all stakeholders within AMCs, including, but not limited to, healthcare providers, public health officials, and researchers, to recognize the elements required to move beyond merely building trust and becoming *trustworthy* to its local community partners.

### Limitations

Despite its innovation, this process had limitations. Though we were able to recruit diverse collaborators and community members from different regions of the country, our sample size was small, and our results may need to be more generalizable. Our study was also limited in that access to broadband and technology (including those living in rural areas) was a critical component of community member participation and thus may have excluded some under-resourced populations, further limiting the generalizability of this study. This also impacts the validity of the product, particularly given the challenges posed by the COVID-19 pandemic for those without broadband access. Finally, due to logistical barriers, we needed to formally assess community members' satisfaction with or reflections on the recruitment and partnership process. Though we received overwhelmingly positive feedback indirectly from CHARGE collaborators, a structured evaluation of participating community members' perceptions would have bolstered the validity of the process and product.

### Future implications

Moving forward, the sustained three-way partnership model will allow for future collaborations with stakeholders that facilitate the refinement and effectiveness of community-academic partnerships that seek to address historical issues of mistrust between AMCs and groups who have been historically marginalized.

Further, in addition to the usefulness of the Toolkit, the PoT remains a hallmark of the Center for Health Justice's work and enjoys ongoing interest and adoption. The AAMC Center for Health Justice is continuing investment in the PoT and has planned implementation, evaluation, and dissemination activities for 2023 and beyond. Further, to address some of the study's limitations, it will be critical to share the toolkit with those community members who did not have broadband internet to validate its ability to represent the lived experiences, beliefs, and circumstances of under-resourced groups. There may also be an opportunity to conduct a similar study post-pandemic with in-person interviews to increase sample inclusivity, as well as disseminate an evaluation of the existing toolkit and partnership by participating community members. Moreover, the PoT Toolkit can be used by AMC leaders to set aside dedicated time to have facilitated discussions within their communities of healthcare providers, researchers, and community stakeholders/members. These discussions will allow all involved in unpacking the principles to explore how they uniquely come to life locally and determine what actions might be taken to demonstrate trustworthiness. Ultimately allowing for enhanced relationship building with broad coalitions, the ability to track lessons learned, and highlighting the importance of health leaders co-creating and sustaining multi-sector community partnerships with the explicit mission to improve population health.

## Data availability statement

The original contributions presented in the study are included in the article/Supplementary material, further inquiries can be directed to the corresponding author.

## Ethics statement

The human subjects' research described in this Innovation Report was approved by American Institutes for Research's Institutional Review Board IRB00000436 on 8/14/20, project EX00530. The patients/participants provided their written informed consent to participate in this study. Written informed consent was obtained from the individual(s) for the publication of any potentially identifiable images or data included in this article.

## Author contributions

PA and KS conceived the original idea and helped supervise the project. KS and OC spearheaded the development of research materials while LA, EA, DT, SM, SG-W, and ML carried out the research. OC and SP oversaw logistics related to research implementation. OC, SP, PA, and KS spearheaded the analysis. All authors discussed the results and contributed to the final manuscript.

## Footnotes

^3^Chapter co-author Lauri Andress' public health website (placeandhealthwv.com) is a qualitative epidemiological profile of inequities using stories, narratives, and images to portray the lived experiences of historically marginalized groups that lie behind the past and present-day rates of death and disease reported in one region of the US.

^4^The development of toolkits began in 2015 with video recorded interviews from a single region soliciting their perspectives on how medical education, clinical care, and research can and should respond to social injustice and the social determinants of health. The resulting videoed interviews were used to frame a conversation during the annual AAMC meeting.

^5^“Trustworthiness” is defined as being worthy of confidence or being responsible, safe, and secure (https://www.merriam-webster.com/dictionary/trustworthiness). Accordingly, trustworthiness is defined in relation to its amplification of the core principles of community engagement, including bi-directionality, shared governance, and inclusivity.
