# Supplementary material for: From the national to the local: Issues of trust and a model for community-academic-engagement
Source: Front Public Health. 2023 Feb 24;11:1068425. doi: 10.3389/fpubh.2023.1068425 (PMC10000727; doi:10.3389/fpubh.2023.1068425)
Supplement: Supplementary file 1 [file Table_1.DOCX]

# Step 1: Define your Community

As a group activity, it’s important to determine: who is your community? Below is a suggestion to get you started.

Review the Community Health Needs Assessments (CHNA).

Most non-profit hospitals, local public health departments and FQHCs must develop a CHNA. You should consider reviewing your community’s various CHNAs as a jumping off point for discussion.

- How was the community defined in each CHNA? Are the definitions aligned?
- How deeply were community members involved in the process?
- How does your organization use these CHNAs to guide its own community-based work?

# Step 2: Define and Discuss Trust in your Community

Next, take the time to talk about trust in your community, between it and your organization. Some questions to consider:

- Assuming mistrust exists in your community, what are the barriers and actions that sow and reinforce mistrust in institutions?
- How does mistrust of institutions lead to decreased confidence in interventions such as vaccinations?

# Step 3: Show the Community Video

After watching the video, have a discussion with your group. Some questions to reflect upon:

- How did the local issues of trust you talked about in Step 2 play out during the COVID19 pandemic?
- Which video themes resonated with your community, which did not?
- Are any themes missing?

For more information about this video, refer to the [Video Guide](#Video_Guide).

# Step 4: Dive into the 10 Principles of Trustworthiness Activities

## Activity One: Show Overview of 10 Principles of Trustworthiness video

Keep in mind that although the Principles are not COVID19 related, their development stems from lessons we re-learned from the pandemic – lessons about trust and community engagement.

For more information about this video, refer to the [Video Guide](#Video_Guide).

## Activity Two: Discover your Community via Appreciative Inquiry

This activity can be conducted with staff or (better still) with staff along with community members. Click the link below for detailed instructions.

### [Appreciative Inquiry Model](#App_Inquiry)

Appreciative Inquiry is a philosophy of relationship building and problem solving. It flips the focus from what isn’t working to what is working, and how to build on that. When the positives of a situation or relationship are highlighted, stakeholders are energized, responses are constructive, and confidence in a strategy for moving forward becomes mutual.

## Activity Three: Work through the Community Engagement Action Guide

The goal of the [Community Engagement Action Guide](#Activities_Template) is to begin moving the 10 Principles of Trustworthiness from concept to action. One or more example activities is suggested for each Principle.

The example activities are presented in grid format, using the following column headings: Questions for Discussion, Your Response to Questions, Suggested Action, and Additional Actions, allowing your group to work through each of the examples.

- You may begin with internal discussion and reflection, and then involve community members. Or you may choose to immediately convene a discussion with community members as you work through the activities
- Space is allowed for your group’s response to the discussion questions, as well as additional actions you might consider in response to the activity.

## Activity Four: Revisit the Outcome of Appreciative Inquiry

Now that you’ve worked through the Community Action Guide, circle back to the takeaways from the Appreciative Inquiry or Appreciative Interviews, and build upon your work, keeping the 10 Principles in mind.

# Step 5: Make use of the Community Engagement Reflection Guide

Community engagement and integration is an iterative, on-going, long-term investment that is foundational to the work of demonstrating trustworthiness. It’s more than building trust in one project or community interaction, but rather building trust in the organization and in the system.

Use the [Community Engagement Reflection Guide](#Reflection_Guide) for personal self-reflection or as a tool to help your organization reflect upon all *10 Principles of Trustworthiness* as you engage with your community.

# Step 6: Further (or Future) Community Engagement Work

Collect information from the Heath Equity Inventory (HEI)

To continue the work of establishing trust with your community, consider making use of the *Health Equity Inventory* to collect information about initiatives across research, education, and clinical care mission areas. The inventory will help you to identify areas of redundancy and opportunities to increase efficiency and impact.

- Health Equity Inventory Instructions

[Download instructions (PDF)](https://www.aamc.org/media/34596/download) on how to enter and analyze data, construct queries, and create reports.

- Health Equity Inventory Spreadsheet

[Download the spreadsheet (Excel)](https://www.aamc.org/media/34636/download) on how to document each of your institution’s community-relevant or health equity-focused initiatives.

**Note**: A new initiative, in the planning stages at the Center for Health Justice, is to expand and enhance the Health Equity Inventory to make it more accessible to a cross-sector audience, beyond Academic Medical Centers (AMCs). The current version of the HEI can, however, be edited to adapt to your organization’s needs.

This work is funded by a cooperative agreement from the Centers for Disease Control and Prevention (CDC): Improving Clinical and Public Health Outcomes through National Partnerships to Prevent and Control Emerging and Re-Emerging Infectious Disease Threats (Award # 1 NU50CK000586-01-00).

# Video Guide: 10 Principles of Trustworthiness

## Community Video

This 11-minute video features interviews with 30 community members from diverse areas across the United States. The interviews were recorded in the summer of 2020, at a time of great tribulation in our society. The video highlights what diverse communities across the country had to say about trust in health care, science, public health, and the COVID19 vaccine.

From those time-bound stories we collectively re-learned lessons about community engagement and trustworthiness. With those lessons in mind, the *10 Principles of Trustworthiness* were extracted from lived experience, and then generalized in recognition that trust isn’t built by developing confidence in one vaccine, but rather by our institutions and organizations acting in ways that demonstrate they are worthy of a community’s trust, whether there is a health crisis underway or not.

The video underscores reasons and causes for mistrust of the health care and other systems, and offers suggested actions that organizations of all kinds can take to demonstrate they are trustworthy. It can be shown as a way to kick off and facilitate a discussion of the 10 Principles of Trustworthiness.

[Community Video: English Version](https://vimeo.com/549008740) [Community Video: Spanish Version](https://vimeo.com/549008885)

Some prompting questions to consider:

1. What was your reaction to the video?
   - Does it resonate with your experiences during the summer of 2020?
   - Does it resonate with any of your experiences outside of the events of 2020?
2. What barriers or actions were brought up that contribute to mistrust of health care and public health in your community?
3. Are there reasons specific to your local community why people might not trust the COVID19 vaccine and other medical interventions?
4. Why might people not trust health “experts?” Whose voices are most valued in your community?
5. What types of actions could your organization take to build trust?

## Overview of the 10 Principles of Trustworthiness

This video could be sent to the audience as pre-work and/or shown as an introduction to the 10 Principles as a way to generate discussion. Some prompting questions to consider:<**Insert link to video**>

1. How might each Principle help address the barriers to mistrust within your local community?

Which principles presented a new and different perspective for your organization?

Which principles could your organization be doing a better job with?

If your organization could choose one or two Principles to focus on right now, what would they be?

# Appreciative Inquiry

Appreciative Inquiry is a philosophy of relationship building and problem solving. It flips the focus from what isn’t working to what is working, and how to build on that. When the positives of a situation or relationship are highlighted, stakeholders are energized, responses are constructive, and confidence in a strategy for moving forward becomes mutual.

## Conducting the Appreciative Inquiry using the 5-D Model

The 5-D model breaks down the activity into discrete steps. Each step involves all participants and can take minutes or hours, depending on the depth of the discussion.

**Define:** participants discuss and define the topic of inquiry. For the Principles of Trustworthiness project, the broad topic is engaging with the community in ways that demonstrate trustworthiness. This step can be further defined and honed by the participating group.

**Discover**: participants explore “the best of what is,” identifying the organization’s strengths, best practices, and sources of excellence, vitality, and peak performance. Provide specific examples as you consider questions such as:

- What has worked best when engaging in your community?
- What are your organization’s strengths?

**Dream**: participants envision a future they really want – a future where the organization is fully engaged and successful around its core purpose and strategic objectives. Consider these questions:

- What does your community really want from a partnership with the organization?
- What does the ideal situation look like when the organization is working and partnering with your community?
- How can you work to integrate your organization into the community rather than engage as an outsider?

**Design**: participants leverage the best of what is, and their visions for the future, to design high-impact strategies that move the organization creatively and decisively in the right direction. Think about and discuss ideas such as the following:

- List the best of the current community/organization’s relationship.
- Envision that relationship in the future. Describe what it looks like in specific detail.
- Environment, setting, location
- Meeting times and location
- Activities you’re both involved in
- Roles within that relationship

**Destiny** (may also be called Deploy): participants put the strategies into action, revising as necessary. They discuss and plan an implementation strategy based on the mutually agreed upon vision from the Design phase.

Source: [Appreciative Inquiry Commons - The Appreciative Inquiry Commons (champlain.edu)](https://appreciativeinquiry.champlain.edu/)

## Conducting the Appreciative Inquiry using the Appreciative Interview Model

[Liberating Structures (Appreciative Interviews)](https://www.liberatingstructures.com/5-appreciative-interviews-ai/) has adapted the 5-D Model, using the interview as a method to more quickly gather information among participants. It is structured in way that enables progress in about an hour.

The following information was taken directly from the **Liberating Structures** website:

In less than one hour, a group of any size can generate the list of conditions that are essential for its success. You can liberate spontaneous momentum and insights for positive change from within the organization as “hidden” success stories are revealed. Positive movement is sparked by the search for what works now and by uncovering the root causes that make success possible.

Groups are energized while sharing their success stories instead of the usual depressing talk about problems. Stories from the field offer social proof of local solutions, promising prototypes, and spread innovations, while providing data for recognizing success patterns. You can overcome the tendency of organizations to underinvest in social supports that generate success, while overemphasizing financial support, time, and technical assistance.

**There are 5 Elements to Appreciative Interview:**

1. Structuring Invitation
   - Ask questions such as:
     - Tell me a story of a time when your community and the organization partnered well on a project or initiative.
     - What made it successful?
   - Have participants partner up in pairs.
2. How Space is Arranged and Materials Needed
   - Unlimited number of groups
   - Chairs for people to sit in pairs face-to-face; no tables needed.
   - Paper for participants to take notes
   - Flip chart to record the stories and assets/conditions
3. How Participation Is Distributed

- Everyone is included
- Everyone has equal time and opportunity to contribute

1. How Groups Are Configured
   - First pairs, then groups of 4.
   - Encourage groups to be diverse
2. Sequence of Steps and Time Allocation
   - Describe the sequence of steps and specify a theme or what kind of story participants are expected to tell. 3 min.
   - In pairs, participants take turns conducting an interview and telling a success story, paying attention to what made the success possible. 7–10 min. each; 15–20 min. total.
   - In groups of 4, each person retells the story of his or her pair partner. Ask participants to listen for patterns in conditions/assets supporting success and to make note of them.
     15 min. for groups of 4.
   - Collect insights and patterns for the whole group to see on a flip chart. Summarize if needed. 10-15 min.
   - Ask, “How are we investing in the assets and conditions that foster success?” and “What opportunities do you see to do more?” Use [1-2-4-All](https://www.liberatingstructures.com/1-1-2-4-all/) to discuss the questions. 10 min.

**Attribution**: Liberating Structure developed by Henri Lipmanowicz and Keith McCandless. Inspired by and adapted from Professor David Cooperrider, Case Western Reserve University, and consultant Dr. Tony Suchman.

# Community Engagement Action Guide

**The tables below contain an interactive approach that can be used as you discuss how to move each of the 10 Principles of Trustworthiness from concept to action.**

- Tables with an **Orange** header contain an example activity to discuss with your group. You may begin with internal discussion and reflection, and then involve community members. Or you may choose to immediately convene a discussion with community members as you work through the activities
  - Space is allowed for your group’s response to the discussion questions, as well as additional actions you might consider in response to the activity.
- Tables with a **Purple** header can be used to add your own activity, prompting questions, and potential actions.

**Principle 1: The community is already educated, that's why it doesn't trust you.**

Words matter. Be mindful of how you frame your relationship. It is not your job to teach to the gaps you assume the community has. Mistrust is a rational response to actual injustice. The community knows what it doesn’t know and will ask when it thinks you have answers it can trust. (This goes for “empowering” the community, too.)

| **Principle 1 Suggested Activity: Consider your institution’s three most recent community communications re: COVID (either about your work in the community or a message directly to your community)** | | | |
| --- | --- | --- | --- |
| **Questions for Discussion** | **Your Response to Questions** | **Suggested Action** | **Additional Actions** |
| - How was your relationship framed? - What unintended messages might you have been delivering? - How (if at all) was the messaging co-developed with the community? - What channels and methods are available for the community to communicate with your organization? | *(this column can be filled in during or after a training session)* | With community partners, co-create a new style guide for community conversations and messages. Also, identify together the most appropriate venues and channels. | *(this column can be filled in during or after a training session)* |
| **Add your own Activity here for Principle 1** *(this table can be filled in during or after a training session)* | | | |
| **Principle 1 Suggested Activity**: | | | |
| **Questions for Discussion** | **Your Response to Questions** | **Suggested Action** | **Additional Actions** |
|  |  |  |  |

**Principle 2: You are not the only experts.**

People closest to injustice are also those closest to the solutions to that injustice. (That is probably not you or your organization and even if it is, there’s a power imbalance.) Listen to people in your community. They have deployed survival tactics and strategies for decades. Centuries, even. Take notes. Co-develop. Co-lead. Share power.

| **Principle 2 Suggested Activity: If you’ve conducted a community needs assessment in the past or are planning one, consider how it was constructed.** | | | |
| --- | --- | --- | --- |
| **Questions for Discussion** | **Your Response to Questions** | **Suggested Action** | **Additional Actions** |
| - What are the unique assets you think you bring to the table AND the unique assets you think others/partners bring? - To what extent did you involve community members in your most recent community assessment? - Was the community involved in co-developing the questions being asked? - If the assessment involved data collection (e.g. qualitative- interviews, focus groups, or quantitative), who actually collected the data? - How (if at all) did you share results with the community? - How do you recognize community contributions to your work? |  | Look at your current practices re: community assessments and list specific actions you can take to increase community involvement.  In addition to community needs, ensure all assessments include a focus on available community assets. |  |
| **Principle 2 Suggested Activity: Consider your most recent formal meeting with community members.** | | | |
| **Questions for Discussion** | **Your Response to Questions** | **Suggested Action** | **Additional Actions** |
| - What format did the meeting take? - Who was involved in setting the agenda? - How much time was one-way, didactic communication? - What was the process for inviting community members? - Where was it held? - How was it advertised? - How were community responses captured and incorporated? |  | Commit to the co-convening of meetings with shared responsibility for agenda development and meeting oversight.  Ensure all communities have an equitable opportunity to attend and participate. |  |
| **Add your own Activity here for Principle 2** *(this table can be filled in during or after a training session)* | | | |
| **Principle 2 Suggested Activity**: | | | |
| **Questions for Discussion** | **Your Response to Questions** | **Suggested Action** | **Additional Actions** |
|  |  |  |  |

**Principle 3: Without action, your organizational pledge is only performance.**

Walk the walk, please. Deploy resources. Coordinate across your organization. Hire someone to the C—suite AND a network/coalition of experts to be responsible because transformation is not a one-person job. Be authentic. Don’t just say you’re committed to the goal of health equity, do the work to achieve it.

| **Principle 3 Suggested Activity: Conduct an evaluation of your community engagement infrastructure.** | | | |
| --- | --- | --- | --- |
| **Questions for Discussion** | **Your Response to Questions** | **Suggested Action** | **Additional Actions** |
| - Does your institution have a dedicated staff position(s) to work with the community? - Does your organization have a formal strategic plan related to your community engaged work? How do you evaluate progress? - To what extent do all staff positions incorporate working with the community? - What are some coordination points between staff and the community? - What internal resources are available to support this work? Are they sufficient? |  | Revisit (or develop) a formal strategic plan related to engagement and trustworthiness. Work with your community partners to identify gaps and required support.  With your community partners, develop metrics of success and the capacity to track them going forward. |  |
| **Add your own Activity here for Principle 3** *(this table can be filled in during or after a training session)* | | | |
| **Principle 3 Suggested Activity**: | | | |
| **Questions for Discussion** | **Your Response to Questions** | **Suggested Action** | **Additional Actions** |
|  |  |  |  |

**Principle 4: An office of community engagement is insufficient.**

One FTE doesn’t cut it. And don’t jam this work into your existing diversity and inclusion office, either. Trustworthiness is not a “minority tax”: we are *all* responsible. This is system-wide, all-hands-on deck work, and as such should be acknowledged, incented, and promoted – literally.

| **Principle 4 Suggested Activity: Review your organization’s policies on tenure and promotion** | | | |
| --- | --- | --- | --- |
| **Questions for Discussion** | **Your Response to Questions** | **Suggested Action** | **Additional Actions** |
| - To what extent is the work being valued by the institution through reward and recognition? - How do you ensure that responsibility for engaging with the community is equitably distributed across your organization? |  | Review and revise merit and promotion policies, and requirements to recognize community service, scholarship and engagement activities. |  |
| **Add your own Activity here for Principle 4** *(this table can be filled in during or after a training session)* | | | |
| **Principle 4 Suggested Activity**: | | | |
| **Questions for Discussion** | **Your Response to Questions** | **Suggested Action** | **Additional Actions** |
|  |  |  |  |

**Principle 5: It doesn’t start or end with a community advisory board.**

Running *your* thoughts by a group of self-appointed community leaders for a thumbs-up does not suffice. Take to the streets to get some unfiltered opinions. And then work together with the community to put that wisdom into the work. Make it clear to all you’ve done so and explain the benefits accrued.

| **Principle 5 Suggested Activity: Closely examine your current advisory board(s).** | | | |
| --- | --- | --- | --- |
| **Questions for Discussion** | **Your Response to Questions** | **Suggested Action** | **Additional Actions** |
| - What was the primary purpose/goal of establishing it? - How was it constructed? - Who’s on it? - How were members selected? - How (if at all) was it announced? - How many Community Advisory Boards (CABs) exist across your organization? Are they complementary? Aligned? How do you know? - What recognition/support do you provide for your advisory members? - How are you evaluating and communicating the impact of the community advisory board? - What language and tone do you use when communicating with your advisory board? - What channels/forums do you use to communicate with your advisory board? |  | Identify and review all CABs across your organization.  Assess composition and internal policies regarding the process of nomination/selection.  Address gaps with an eye toward diversity, inclusion, and new perspectives. |  |
| **Add your own Activity here for Principle 5** *(this table can be filled in during or after a training session)* | | | |
| **Principle 5 Suggested Activity**: | | | |
| **Questions for Discussion** | **Your Response to Questions** | **Suggested Action** | **Additional Actions** |
|  |  |  |  |

**Principle 6: Diversity is more than skin deep.**

We are diverse within our diversity. Do not rely solely on matching skin tones to make a difference. Think intersectionality and multiple identities but remember: humility and honesty are the foundation for earning trust.

| **Principle 6 Suggested Activity: Examine your currently active projects that focus on the health of communities that have been under-resourced and minoritized.** | | | |
| --- | --- | --- | --- |
| **Questions to for Discussion** | **Your Response to Questions** | **Suggested Action** | **Additional Action** |
| How do you identify and select staff who are asked to serve on diversity and heath equity related groups, task forces, etc.?  Who is leading them?  How did they get there?  Do you see any patterns?  Are there other experts that are not engaged in the work? Why not? |  | Convene your organization’s current community engagement leaders and review the Discussion Questions with them.  Develop a plan to broaden organizational expectations about who can and should lead this work. |  |
| **Add your own Activity here for Principle 6** *(this table can be filled in during or after a training session)* | | | |
| **Principle 6 Suggested Activity**: | | | |
| **Questions for Discussion** | **Your Response to Questions** | **Suggested Action** | **Additional Actions** |
|  |  |  |  |

**Principle 7: There's more than one gay bar, one “Black church” and one bodega in your community.**

Not all gay people go to the club, and not all people of color go to the same church (or go at all.) Know *all* of your community’s assets. Visit them. Meet the patrons. Meet the leaders. Break bread and share a meal – at their tables.

Note: For more information on activities, including the Health Equity Inventory, please see [Building a Systems Approach to Community Health and Health Equity | AAMC](https://www.aamc.org/what-we-do/mission-areas/medical-research/health-equity/systems-approach)

| **Principle 7 Suggested Activity: Research the resources available in your local community.** | | | |
| --- | --- | --- | --- |
| **Questions for Discussion** | **Your Response to Questions** | **Suggested Action** | **Additional Actions** |
| Has your organization’s leadership visited community partners’ sites?  Do clinicians visit the community organizations to which they're referring patients for social services?  What is your strategy for ensuring broad engagement across your community?  What have you learned from residents about the community's assets and resources?  What opportunities exist for people from your organization to spend real time with the community at events that matter to the community? |  | Use the Health Equity Inventory (or another data collection method) to review which community partners might be overburdened and which might be under-engaged.  Develop a strategy and action steps to broaden your community connections. |  |
| **Add your own Activity here for Principle 7** *(this table can be filled in during or after a training session)* | | | |
| **Principle 7 Suggested Activity**: | | | |
| **Questions for Discussion** | **Your Response to Questions** | **Suggested Action** | **Additional Actions** |
|  |  |  |  |

**Principle 8: Show your work.**

The community does not think you are perfect, and the past is always present. So be transparent with your limitations, your biases, your goals, your funding, and the outcomes that matter to you. Then ask the community to do the same. Identify the “win-win” for all parties. No secrets, no surprises.

| **Principle 8 Suggested Activity: Take a close look at one of your most recently funded community projects and the outcomes** | | | | | | |
| --- | --- | --- | --- | --- | --- | --- |
| **Questions for Discussion** | **Your Response to Questions** | | **Suggested Action** | | | **Additional Actions** |
| - How do you ensure that all players in the programs/projects have aligned goals and objectives? - Did you share information regarding the allocation and source of funding? - Did you discuss/co-create how outcomes would be evaluated and disseminated? - How are you setting up programs/projects to ensure sustainability? - What are your policies on sharing data? |  | | Develop and incorporate consensus-building processes for your next set of programs/projects. | | |  |
| **Add your own Activity here for Principle 8** *(this table can be filled in during or after a training session)* | | | | | | |
| **Principle 8 Suggested Activity**: | | | | | | |
| **Questions for Discussion** | | **Your Response to Questions** | | **Suggested Action** | **Additional Actions** | |
|  | |  | |  |  | |

**Principle 9: If you’re gonna do it, take your time, do it right.**

Demonstrating trustworthiness is not a one-and-done proposition. Keep at it. Be mindful. And remember, it takes a long time to build trust and only a split second to destroy it. Pace yourself.

| **Principle 9 Suggested Activity: Plot the life course of a typical community engaged project for your org and then think through ways to improve it.** | | | |
| --- | --- | --- | --- |
| **Questions for Discussion** | **Your Response to Questions** | **Suggested Action** | **Additional Actions** |
| - How does your community assess the strength of its partnership with you? What tools does it use? - How do you assess the strength of your community partnerships? What tools do you use? - Reflect on partnerships that you think are well developed. What characteristics do they have? - How long did they take to build? - Why are they successful? - What doesn’t work? |  | Create a roadmap: short-term and longer-term commitments, including key milestones and ongoing assessments.  Co-develop with community partners mechanisms for gathering feedback from the community on an ongoing basis. |  |
| **Add your own Activity here for Principle 9** *(this table can be filled in during or after a training session)* | | | |
| **Principle 9 Suggested Activity**: | | | |
| **Questions for Discussion** | **Your Response to Questions** | **Suggested Action** | **Additional Actions** |
|  |  |  |  |

**Principle 10: The project may be over, but the work is not.**

Do not drop in and drop out. Share results. Partner on next steps. Close the loop. The community is constant – it is not there only for the duration of your grant or initiative. Be there for it always and it is more likely to want to be there for you.

| **Principle 10 Suggested Activity: Reflect on a recent community project that ended more than 1 year ago.** | | | |
| --- | --- | --- | --- |
| **Questions for Discussion** | **Your Response to Questions** | **Suggested Action** | **Additional Actions** |
| - To what extent were community members co-leaders of the work? - To what extent were community members involved in a two-way dialogue about the results and findings? - How are bidirectional communication channels maintained over time, even in the absence of a specific work? - How can the community best engage with you? - How do you plan to continue engagement after a current project? |  | Develop a sustainability model for the community to continue the work that's important to them, with your continued support and engagement.  With community partnership, create routine opportunities for connection and relationship building, outside of projects and work settings. |  |
| **Add your own Activity here for Principle 10** *(this table can be filled in during or after a training session)* | | | |
| **Principle 10 Suggested Activity**: | | | |
| **Questions for Discussion** | **Your Response to Questions** | **Suggested Action** | **Additional Actions** |
|  |  |  |  |

# Community Engagement Reflection Guide

| **10 Principles of Trustworthiness** |
| --- |
| Principle 1 – The community is already educated, that's why it doesn't trust you. **Key Behavior: Respect and Responsibility**  How are we communicating with the community as a dialogue between equals, instead of a one-way lesson?  How do we take the time to understand local knowledge, history and its ramifications?   - How do we ask the community what is needed rather than assume? |
| Principle 2 – You are not the only experts. **Key Behavior: Humility**   - How are you using language that conveys superiority or an “us versus them” mentality? - In what ways are you partnering or trying to “lead” the coalition? - Can we point to concrete ways you’re incorporating community expertise into your work? |
| Principle 3 – Without action, your organizational pledge is only performance. **Key Behavior: Authenticity**   - How are we following up our words with meaningful action? - How does our plan for action hold organizational leadership accountable? - How is our plan for action and related evaluation co-developed with the community? |
| Principle 4 – An office of community engagement is insufficient. **Key Behavior: Commitment**   - Have we clearly communicated organization-wide policies and expectations for working with the community? - How have we demonstrated an understanding across the organization that this work is valuable? - Do we have a strategy that reflects how the work aligns with the assets, needs, and goals of the community? - Do we have financial resources to adequately pay community stakeholders for their work? |
| Principle 5 – It doesn’t start or end with a community advisory board.  **Key Behavior: Diversity of Thought**   - How are we engaging our community beyond the Community Advisory Board (CAB)? - How are we working **within** the community in different ways? - Who is missing from your CAB? Why? |
| Principle 6 – Diversity is more than skin deep.  **Key Behavior: Intersectionality**   - In what ways are we supporting honest communication within our internal groups and communities? - How does our engagement approach consider that diversity is more than just race and ethnicity? - How have we created an engagement approach that both understands and values the multiple identities and lived experiences of community members? |
| Principle 7 – There's more than one gay bar, one “Black church” and one bodega in your community**.**  **Key Behavior: Relationship Building**   - Do we routinely engage the same community organizations and leaders in our organization’s community engagement work? Who is missing? - In what ways are we getting out to meet and interact with community members where they work and live? |
| Principle 8 – Show your work.  **Key Behavior: Transparency**   - How do we demonstrate clarity and intentionality about working **with** the community? - Are we co-developing expectations and goals with the community? - Are we creating an open, honest, and accessible environment where community members feel safe and are able to provide feedback? |
| Principle 9 – If you’re gonna do it, take your time, do it right**.**  **Key Behavior: Intentionality**   - How are we continuing to build and maintain trust within the community? - Are members of the community co-developing, and involved in the continuous improvement cycle of the tools that support their community? |
| Principle 10 – The project may be over, but the work is not.  **Key Behavior: Maintaining the Relationship**   - Is there a plan to engage the community even after the project or initiative ends? - How are evaluation and dissemination plans built into the partnership with the community? - In what ways do we engage our community partners when not tied to a specific project? |
