# Supplementary material for: From the national to the local: Issues of trust and a model for community-academic-engagement
Source: Front Public Health. 2023 Feb 24;11:1068425. doi: 10.3389/fpubh.2023.1068425 (PMC10000727; doi:10.3389/fpubh.2023.1068425)
Supplement: Supplementary file 3 [file Data_Sheet_1.PDF]

AAMC LSL 2020 Session: *Communities, Social Justice, and Academic Medical Centers*
Community Interview Guide

INTRODUCTION: Hi. Today we'd like to ask you a few questions about how you think an academic medical center can improve your health and the health of your community and your neighborhood. An academic medical center does three main things – it provides health care – doctors, nurses, emergency room – it teaches medical students and trains physicians, and it conducts scientific research into how to make people, patients and communities healthier. The goal of our asking you these questions is to educate health professionals and to maybe influence how they are trained and provide care based on your ideas and perspectives.

<u>COMMUNITY</u>

Q0: What do you think of as being your "community"?

- PROMPT if necessary: Is it your city, your neighborhood, school, where you work, church, friends, family, or something else?

Q0.1: Describe the health of your community [insert reply for Q0].

Q1: What would a healthy community look like to you?

Q1.1: How do you feel where you live affects your health?

Q1.2: How do you feel like other factors such as your race, culture, gender, education level, personal finances, or anything else we didn't mention, affect your health?

<u>CLINICAL CARE</u>

PROMPT: Earlier you talked about how where you live and other factors affect your health. You talked about [insert responses from Q1.2].

Q2: How do these issues come up during your doctor visits?

[If respondent <u>DOESN'T</u> discuss Q1.2 with doctor, then Q3]
[If respondent <u>DOES</u> discuss Q1.2 with doctor, then Q4]

Q3: Why don't you talk about those things with your doctor? [Skip to Q5]

Q4. When you discuss those concerns with your doctor what does your doctor do? How does he or she respond?

Q5: What do you think doctors should be doing about the things happening in your community that affect your health?

MEDICAL EDUCATION

PROMPT: Earlier I mentioned that in addition to caring for patients, academic medical centers teach students and train doctors.

Q6: What do you think medical students should be taught about the strengths and challenges of your community?

Q7: What's one thing you think medical students should know about the health of your community?

RESEARCH

PROMPT: In addition to teaching and training doctors, academic medical centers also teach and train professionals who conduct research to improve the lives of patients and communities. The kind of research we're talking about could be a scientist in a lab working with microscopes, it could be research into whether a new medication works better than the old one, and it could be research in the healthcare system or the community looking at whether a program to improve health works.

Q8: Have you ever participated in a research study (this could be a clinical trial, an opinion survey, etc.)?

Q9: Would you participate in a clinical trial to help find a COVID-19 vaccine?

[If respondent says YES, then Q9.1]
[If respondent says NO, then Q9.2]

Q9.1: Can you tell me why you said yes?

Q.9.2: Can you tell me why you would decline?

Q10: I know you said [insert response to Q9]. How do you think other people from your community would feel about participating in vaccine research or any other type of research?

Q11: Research is often improved when done in partnership with communities. How can academic medical centers better work with their local communities?
