# Supplementary material for: From the national to the local: Issues of trust and a model for community-academic-engagement
Source: Front Public Health. 2023 Feb 24;11:1068425. doi: 10.3389/fpubh.2023.1068425 (PMC10000727; doi:10.3389/fpubh.2023.1068425)
Supplement: Supplementary file 4 [file Data_Sheet_2.PDF]

Community Engagement Reflection Guide

AAMC
CENTER FOR HEALTH JUSTICE

Community engagement is an iterative, on-going, long-term investment that is foundational to the work of demonstrating trustworthiness. It's more than building trust in one project or community interaction, but rather building trust in the organization and in the system.

Use this guide for personal self-reflection or as a tool to help your organization reflect upon all *10 Principles of Trustworthiness* as you engage with your community.

10 Principles of Trustworthiness

Principle 1 – The community is already educated, that's why it doesn't trust you.

Key Behavior: Respect and Responsibility

- How are we communicating with the community as a dialogue between equals, instead of a one-way lesson?
- How do we take the time to understand local knowledge, history and its ramifications?
- How do we ask the community what is needed rather than assume?

Principle 2 – You are not the only expert.

Key Behavior: Humility

- How are you using language that conveys superiority or an "us versus them" mentality?
- In what ways are you partnering or trying to "lead" the coalition'?
- Can you point to concrete ways you're incorporating community expertise into your work?

Principle 3 – Without action, your organizational pledge is only performance.

Key Behavior: Authenticity

- How are we following up our words with meaningful action?
- How does our plan for action hold organizational leadership accountable?
- How is our plan for action and related evaluation co-developed with the community?

Principle 4 – An office of community engagement is insufficient.

Key Behavior: Commitment

- Have we clearly communicated organization-wide policies and expectations for working with the community?
- How have we demonstrated an understanding across the organization that this work is valuable?
- Do we have a strategy that reflects how the work aligns with the assets, needs, and goals of the community?
- Do we have financial resources to adequately pay community stakeholders for their work?

Principle 5 – It doesn't start or end with a community advisory board.

Key Behavior: Diversity of Thought

- How are we engaging our community beyond the Community Advisory Board (CAB)?

© 2021 Association of American Medical Colleges

10 Principles of Trustworthiness

- How are we working **within** the community in different ways?
- Who is missing from your CAB? Why?

Principle 6 – Diversity is more than skin deep.

Key Behavior: Acknowledge Intersectionality

- In what ways are we supporting honest communication within our internal groups and communities?
- How does our engagement approach consider that diversity is more than just race and ethnicity?
- How have we created an engagement approach that both understands and values the multiple identities and lived experiences of community members?

Principle 7 – There's more than one gay bar, one "Black church" and one bodega in your community.

Key Behavior: Relationship Building

- Do we routinely engage the same community organizations and leaders in our organization's community engagement work? Who is missing?
- In what ways are we getting out to meet and interact with community members where they work and live?

Principle 8 – Show your work.

Key Behavior: Transparency

- How do we demonstrate clarity and intentionality about working **with** the community?
- Are we co-developing expectations and goals with the community?
- Are we creating an open, honest, and accessible environment where community members feel safe and are able to provide feedback?

Principle 9 – If you're gonna do it, take your time, do it right.

Key Behavior: Intentionality

- How are we continuing to build and maintain trust within the community?
- Are members of the community co-developing, and involved in the continuous improvement cycle of the tools that support their community?

Principle 10 – The project may be over, but the work is not.

Key Behavior: Maintaining the Relationship

- Is there a plan to engage the community even after the project or initiative ends?
- How are evaluation and dissemination plans built into the partnership with the community?
- In what ways do we engage our community partners when not tied to a specific project?

This work is funded by a cooperative agreement from the Centers for Disease Control and Prevention (CDC): Improving Clinical and Public Health Outcomes through National Partnerships to Prevent and Control Emerging and Re-Emerging Infectious Disease Threats (Award # 1 NU50CK000586-01-00)
