# Supplementary material for: From the national to the local: Issues of trust and a model for community-academic-engagement
Source: Front Public Health. 2023 Feb 24;11:1068425. doi: 10.3389/fpubh.2023.1068425 (PMC10000727; doi:10.3389/fpubh.2023.1068425)
Supplement: Supplementary file 5 [file Data_Sheet_3.PDF]

AAMC Principles of Trustworthiness

AAMC
CENTER FOR HEALTH JUSTICE

Deserving trust is crucial to equitably partner with the communities you engage and to achieve health justice.

Remember, though, **the process of engagement is as important as the product.** Here are 10 principles that community stakeholders endorse as the guiding compass on your journey to establishing trustworthiness.

1 The community is already educated; that's why it doesn't trust you.

Words matter. Be mindful of how you frame your relationship. It is not your job to teach to the gaps you assume the community has. Mistrust is a rational response to actual injustice. The community knows what it doesn't know and will ask when it thinks you have answers it can trust. (This goes for "empowering" the community, too.)

2 You are not the only experts.

People closest to injustice are also those closest to the solutions to that injustice. (That is probably not you or your organization and, even if it is, there's a power imbalance.) Listen to people in your community. They have deployed survival tactics and strategies for decades — centuries, even. Take notes. Co-develop. Co-lead. Share power.

3 Without action, your organizational pledge is only performance.

Walk the walk, please. Deploy resources. Coordinate across your organization. Hire someone to the C-suite *and* a network or coalition of experts to be responsible for transformation because transformation is not a one-person job. Be authentic. Don't just say you're committed to the goal of health equity; do the work to achieve it.

4 An office of community engagement is insufficient.

One full-time employee doesn't cut it. Don't jam this work into your existing diversity and inclusion office, either. Trustworthiness is not a "minority tax"; we are *all* responsible. This is systemwide, all-hands-on-deck work and, as such, should be acknowledged, incentivized, and promoted in material ways.

5 It doesn't start or end with a community advisory board.

Running *your* thoughts by a group of self-appointed community leaders for a thumbs-up does not suffice. Take to the streets to get some unfiltered opinions. And then work together with the community to put that wisdom into the work. Make it clear to all you've done so, and explain the benefits accrued.

6 Diversity is more than skin deep.

We are diverse within our diversity. Do not rely solely on matching skin tones to make a difference. Think intersectionality and multiple identities, but remember: humility and honesty are the foundation for earning trust.

7 There's more than one gay bar, one "Black church," and one bodega in your community.

Not all gay people go to the club, and not all people of color go to the same church (or go at all). Know *all* of your community's assets. Visit them. Meet the patrons. Meet the leaders. Break bread and share a meal — at their tables.

8 Show your work.

The community does not think you are perfect, and the past is always present. So be transparent about your limitations, your biases, your goals, your funding, and the outcomes that matter to you. Then ask the community to do the same. Identify the "win-win" for all parties. No secrets, no surprises.

9 If you're gonna do it, take your time, do it right.

Demonstrating trustworthiness is not a one-and-done proposition. Keep at it. Be mindful. Remember, it takes a long time to build trust and only a split second to destroy it. Pace yourself.

10 The project may be over, but the work is not.

Do not drop in and drop out. Share results. Partner on next steps. Close the loop. The community is constant — it is not there only for the duration of your grant or initiative. Be there for the community, always, and it is more likely to want to be there for you.

AAMC Principles of Trustworthiness

Resources

Ahmed S, Neu Young S, DeFino M, Franco Z, Nelson D. Towards a practical model for community engagement: advancing the art and science in academic health centers. *J Clin Transl Res*. 2017;1(5):310-315. doi:10.1017/cts.2017.304.

Andress L, Hall T, Davis S, Levine J, Cripps K, Guinn D. Addressing power dynamics in community-engaged research partnerships. *J Patient Rep Outcomes*. 2020;4:1-8.

Brown AF, Ma GX, Miranda J, et al. Structural interventions to reduce and eliminate health disparities. *Am Journal Public Health*. 2019;109(S1):S72-S78. doi:10.2105/AJPH.2018.304844.

Chung B, Jones L, Dixon EL, et al. Using a community partnered participatory research approach to implement a randomized controlled trial: planning community partners in care. *J Health Care Poor Underserved*. 2010;21(3):780-95.

Clinical and Translational Science Awards Consortium Community Engagement Key Function Committee Task Force on the Principles of Community Engagement. *Principles of community engagement*. Washington, DC: National Institutes of Health, Centers for Disease Control and Prevention, Agency for Toxic Substances and Disease Registry;2011. www.atsdr.cdc.gov/communityengagement/pdf/PCE_Report_508_FINAL.pdf.

Eder MM, Carter-Edwards L, Hurd TC, Rumala BB, Wallerstein N. A logic model for community engagement within the Clinical and Translational Science Awards consortium: can we measure what we model? *Acad Med.* 2013;88(10):1430-1436. doi:10.1097/ACM.0b013e31829b54ae.

Hicks S, Duran B, Wallerstein N, et al. Evaluating community-based participatory research to improve community-partnered science and community health. *Prog Community Health Partnersh*. 2012;6(3):289-299. doi:10.1353/cpr.2012.0049.

Jones L, K Well. Strategies for academic and clinician engagement in community-participatory partnered research. *JAMA*. 2007;297(4):407-10.

Wilkins CH. Effective engagement requires trust and being trustworthy. *Medical Care.* 2018;56(10 Suppl 1):S6-S8. doi:10.1097/MLR.0000000000000953.

Collaborators

Etsemaye P. Agonafer, MD, MPH, MS
Assistant Professor, Department of Health System Science
Kaiser Permanente Bernard J. Tyson School of Medicine

Ben Altenberg
Producer
Readily Apparent Media

Lauri Andress, MPH, JD, PhD
Assistant Professor
Department of Health Policy, Management, and Leadership
West Virginia University School of Public Health

Jennifer Bushelle-Edghill, PhD
Associate Professor
Fayetteville State University

Desiree de la Torre, MPH, MBA
Children's National Hospital
Director, Community Affairs and Population Health Improvement
Child Health Advocacy Institute

Bola F. Ekezue, PhD
Assistant Professor
Fayetteville State University

Shannon Guillot-Wright, PhD
Assistant Professor and Ob/Gyn
Director of Health Policy Research, Center for Violence Prevention
University of Texas Medical Branch

Damon P. Leader Charge
Sicangu Oglala Lakota
Director of Tribal Outreach
University of South Dakota

Marshala Lee, MD, MPH
Harrington Value Institute Community Partnership Director
ChristianaCare

Susan Massick, MD
Associate Professor of Clinical Medicine, Division of Dermatology
The Ohio State University College of Medicine

Namrata Walia
Postdoctoral Research Fellow
Family and Community Medicine
Baylor College of Medicine

Maranda Ward, EdD, MPH
Assistant Professor
The George Washington University School of Medicine and Health Sciences

21/042B (04/21)
